# 

**Published:** 1895-07

**Authors:** 


### CONTENTS:

### SELECTIONS.

Article I.—Amalgam—Its Use from a Practical Standpoint. By Dr. W. E. Hasley. - - - - - - 97
" II.—Bleaching Teeth by Electricity. By Dr. Albert Westlake, - - - - - - - 101
" III.—The Dental Laboratory, - - - - - 103
" IV.—Simple and Complicated Regulating Mechanisms; Æsthetic Mechanisms; Proper and Improper Anchorage. By J. N. Farrar, M. D., D. D. S. - 111
" V.—How to Vulcanize Rubber Plates Between Metallic Surfaces. By Dr. A. N. Dick, - - - - 114
" VI.—Carving of Block-Teeth. By F. A. Coney, D. D. S. 116
" VII.—Rubefacients and Vesicants. By A. W. Harlan, M. D., D. D. S., - - - - - - - 121
" VIII—Attitude of the Dentist Towards His Patient. By R. G. McLaughlin, D. D. S. - - - - - 124
" IX—Will the Too Prevalent Use of Plastics in Filling Teeth Work Injury to Operative Dentistry. By J. D. Patterson, D. D. S. - - - - - 129
" X—Another Method of Banding a Logan Crown, - 134

### COMMUNICATED.

American Dental Association Meeting. - - - - - 136
Odontological Society of Chicago. - - - - - - 137
American Dental Association. - - - - - - - 138

### OBITUARY.

Augustus Woodruff Brown, D. D. S. - - - - - - 139

### EDITORIAL, ETC.

Physicians on the Canadian Border. - - - - - - 140

### MONTHLY SUMMARY.

The Administration of Chloroform. - - - - - - 141

The Influence of Pregnancy on Caries. - - - - - 142

The Sanitary Sin. - - - - - - - - 143

Antral Empyema of Tuberculous Origin. - - - - - 144

Dead Teeth. - - - - - - - - - 144

